# The Level of Oxidative Neutrophil Response When Determining Endotoxin Activity Assay: A New Biomarker for Defining the Indications and Effectiveness of Intensive Care in Patients with Sepsis

**DOI:** 10.1155/2017/3495293

**Published:** 2017-04-12

**Authors:** Michael Yaroustovsky, Ekaterina Rogalskaya, Marina Plyushch, Ludmila Klimovich, Nataliya Samsonova, Marina Abramyan

**Affiliations:** Federal State Budget Institution “Bakulev National Scientific and Practical Center for Cardiovascular Surgery” of the Ministry of Healthcare of the Russian Federation (Bakulev NSPCCS), Moscow, Russia

## Abstract

*Background*. To analyse the clinical informativity of the neutrophil oxidative response level (“Response”) during an Endotoxin Activity Assay (EAA) as a new biomarker defining the indications and effectiveness of intensive care in cardiac surgical patients with septic complications.* Methods*. Blood samples were taken from 198 adult patients who were admitted to the ICU after cardiac surgery (SIRS: 34, MODS: 36, and sepsis: 128). The composite of laboratory studies included CRP, PCT, EAA with “Response” level, and presepsin.* Results*. 83% of patients had a “normal” neutrophil response, 12% of patients had a low neutrophil response, and 5% of patients had a critically low neutrophil response. Patients with critically low responses had the lowest values of the EAA and the highest concentrations of PSP and D-dimer (*p* < 0.05).* Conclusions*. EAA results should be interpreted with the level of neutrophil response. “Response” > 0.5 has a negative predictive value; the EAA < 0.6 at “Response” < 0.5 may indicate a high level of endotoxaemia.

## 1. Background

Sepsis is a precarious public health problem worldwide. Despite significant advances in modern medicine, the mortality from sepsis is still high [1–3]. A risk factor for patients in surgical clinics is the surgery itself, due to the often reduced bacterial load threshold. The incidence of infectious complications in cardiac surgical patients admitted to the ICU varies from 3.7 to 39 cases per 100,000 population, and hospital mortality among these patients can reach up to 79% [4, 5]. A ten-year structural analysis of nosocomial infectious agents conducted at A.N. Bakulev the NSPCCS revealed an increase in the proportion of Gram-negative bacteria, which corresponds to the modern world trends [6, 7]. The most powerful molecule determining the antigenic properties of Gram-negative bacteria is the endotoxin (lipopolysaccharide, LPS), which is a part of the outer cell wall of Gram-negative bacteria [8]. The body's response to the endotoxin is characterized by the release of cytokines and other inflammatory mediators, which leads to the formation of a generalized inflammatory response. Mononuclear and endothelial cells that are activated by the endotoxin induce the expression of platelet-activating factors and coagulation proteases induce additional proinflammatory stimuli. A mutual reinforcement of inflammatory and coagulation cascades leads to tissue damage and the development of septic shock [9, 10].

In the past decade, selective LPS-adsorption that requires a precise definition of the level of endotoxaemia has been proven to be successfully applicable in the complex treatment of sepsis [11–13]. Today, the Medline bibliographic database contains about a hundred publications that mention Endotoxin Activity Assay (ЕАА, Spectral Diagnostics, Toronto, Canada). ЕАА is based on the reaction between the endotoxin in the patient's whole blood and the antiendotoxin antibodies contained in the reagent. Complement proteins opsonize antibody/endotoxin complexes. Through the agency of the receptors being complementary to the complement components, opsonized immune complexes interact with neutrophils that undergo a respiratory burst in the presence of zymosan. Neutrophil oxidants react with luminol. The intensity of chemiluminescence is proportional to the endotoxin level in a patient sample [14, 15]. ЕАА has a special selection, known as «Response», that reflects the degree of neutrophil oxidative response adequacy. The “Response” level is not an individual test. It is an additional parameter of the EAA test. The level of EAA depends on the level of the “Response.” According to comments from the ЕАА manufacturer, a reference range of the «Response» indicator is 0.80–0.98. However, none of the previously published works contained data on the neutrophil “Response” (http://www.ncbi.nlm.nih.gov/pubmed) when implementing the ЕАА in septic patients.

The aim of this study was to analyse the clinical informativity of the neutrophil oxidative response level (“Response”) during the Endotoxin Activity Assay as a new biomarker defining the indications and effectiveness of intensive care in cardiac surgical patients with septic complications.

## 2. Methods

A single-centre prospective study from November 2010 to November 2014 was conducted in 198 patients aged 18 to 78 years who were admitted to the A.N. Bakulev NSPCCS intensive care unit. The study was approved by the local ethics committee of the A.N. Bakulev NSPCCS.

The primary diagnosis of the study population was valvular pathology and/or chronic ischaemic heart disease. According to the New York Heart Association (NYHA) functional classification, 2% of patients were in class I, 14% of patients were in class II, 65% of patients were in class III, and 19% of patients were in class IV. All patients underwent cardiac bypass surgeries with moderate hypothermia and cardioplegia. Most patients (87%) had been operated on for valvular heart disease. Repeat surgical correction was performed in 21 patients. The postoperative period was complicated by the development of low cardiac output syndrome (LVEF < 40%). Haemodynamic maintenance required the use of cardiotonic support with two or more sympathomimetic agents, and in some cases (11%), intra-aortic balloon counterpulsation was applied. Respiratory failure, accompanied by the deterioration of blood gas composition, required prolonged mechanical ventilation in all patients.

The inclusion criteria were age of 18 years or older, the presence of two or more signs of SIRS according to the ACCP/SCCM criteria, and a procalcitonin (PCT) level more than 0.5 ng/mL in the postoperative period. We recorded age, gender, body weight, surgical parameters (duration of aortic cross-clamping and AC), haemodynamic parameters, and severity of illness and organ dysfunction using APACHE II and SOFA scores (Table 1).

The study enrolled 34 (17.2%) patients with the symptoms of SIRS, 36 (18.2%) patients with multiple organ dysfunction syndrome, and 128 (64.6%) patients with sepsis. Most patients with sepsis (86%) had ventilator-associated pneumonia, which was confirmed by clinical data and X-ray results. Mediastinitis and acute pancreatitis were observed in 14 and 10 cases, respectively, and 3 patients had peritonitis due to colonic perforation. Bacteriological examination of bronchoalveolar lavage fluid discovered Gram-negative microorganisms in 61% of patients with septic complications (*Klebsiella pneumoniae*,* Acinetobacter baumannii*, and* Pseudomonas aeruginosa*). Bacteriological examination of blood identified Gram-negative microorganisms in 25% of cases.

The array of laboratory studies included a haematological analysis, an evaluation of haemostasis activation markers, a CRP, and a PCT. Along with the basic tests, the method of chemiluminescence was implemented to measure the EAA (Endotoxin Activity Assay, Spectral Diagnostics) and the presepsin (PSP, Mitsubishi Chemical Medience Corp.) activity levels in patients. In accordance with the inclusion criteria, the levels of the markers were measured on the 7th postoperative day on average.

Selective LPS-adsorption procedures using Toraymyxin-PMX-F columns (Toray) were included in the complex therapy of 66 septic patients due to their clinical condition and a high level of endotoxaemia. These patients were studied clinically and through the use of laboratory values before the first LPS-adsorption procedure and 48 hours after the beginning of the extracorporeal therapy. The other patient group was provided with conventional conservative treatment according to the guidelines of the “Surviving Sepsis Campaign” [16].

The results were statistically assessed with SPSS for Windows version 20.0 (IBM Corp.) using nonparametric statistics. The data are presented as medians and interquartile ranges. The critical level of significance was set at 0.05 [17, 18].

## 3. Results

**Figure 1:**
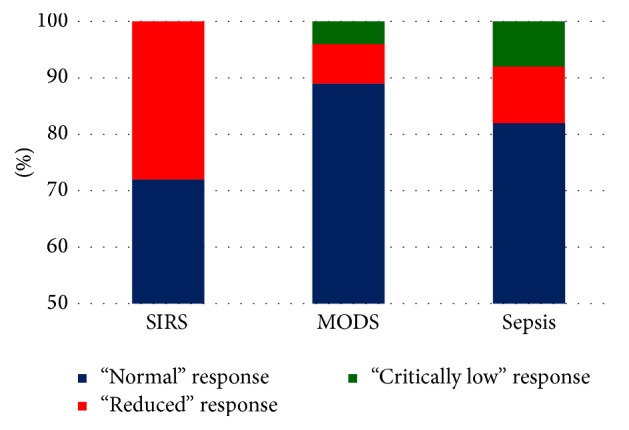
The level of “Response” in patients with different diagnosis.

The «Response» medians of the examined patients were at the level of 0.92 (0.84; 0.95), which corresponded to a normal functional ability of neutrophils. However, parameter fluctuations were observed within the allowable range that extended from 0.01 to 0.98. We have analysed the frequency of occurrence of low and critically low neutrophil response. The “reduced” level of response was recorded when the parametric value amounted to more than 50% of the possible value but remained lower than the recommended minimum limit of the reference range of 0.79–0.50, with values less than 0.50 being considered “critically low.” The «Response» values that corresponded to a “normal” or a reduced functional capacity of neutrophils were observed in the SIRS patients. The highest frequency of a “normal” cellular response was detected in patients that had multiple organ failure. The critically low «Response» was mostly found in septic patients (Figure 1).

The data obtained were analysed by dividing the patients (*n* = 198) into 3 groups: group 1, patients with a “normal” neutrophil response (83%); group 2, patients with a reduced neutrophil response (12%); and group 3, patients with a critically low neutrophil response (5%). The «Response» values were compared to the endotoxin activity and the concentration of inflammatory markers.

The data analysis (Table 2) showed the lowest values of EAA, CRP, PCT, and WBC in the patients from group 3. EAA is not similar between the three levels of the “Response,” but there is a nonsignificant difference (*p* = 0.07) due to the rare frequency (5%) of the patients with critical low level of the “Response.” The concentration of PSP in this group of patients exceeded the concentration of this indicator in groups 1 and 2, by 4.6 and 5.5 times, respectively (p gr1/gr3 = 0.01; p gr2/gr3 = 0.05), and the D-dimer level was 3.3 and 4.4 times higher (p gr1/gr3 = 0.05; p gr2/gr3 = 0.04).

The manifestation of neutrophil respiratory burst capacity is one of the ways that bactericidal properties are implemented that result from the activation of phagocytosis. Since phagocytosis of opsonized foreign substances is peculiar not only for neutrophils but also for other leukocyte subpopulations, this study evaluated the relative number of these cells in groups with different «Response» levels. There were no statistically significant differences in the ratio of phagocytic leukocyte subpopulations among the analysed groups (Table 3).

The high level of EAA (≥0.6) is the indication for the LPS-adsorption. We had no difficulties when using the LPS-adsorption in septic patients with high level of endotoxaemia. The level of EAA was more than 0.60 (Table 4).

There are patients with low level of EAA. These patients may have higher level of endotoxaemia, but the EAA test cannot show it due to the low neutrophil reactivity. These patients also need selective LPS-adsorption. We cannot make a decision using an individual biomarker. We need a panel of biomarkers.

Dynamics of these inflammatory markers and the «Response» parameter is presented within the observed clinical case of patient M., aged 62 years, who developed postoperative Gram-negative sepsis due to ventilator-associated pneumonia (BAL,* Klebsiella pneumoniae*). During the complex therapy for sepsis, the patient received two LPS-adsorption procedures. Endotoxin activity levels were measured to monitor the level of endotoxaemia. Whereas the neutrophil response was only 1%, the EAA value was 0. The observed blood concentrations of CRP, PCT, PSP, and D-dimer were greater than the reference values (Table 5).

After LPS-adsorption procedures, the levels of neutrophil oxidative burst capacity and endotoxin activity increased, PSP and D-dimer concentrations reduced, total WBC counts reached the normal range, and CRP and PCT increased slightly. We observed a decrease in body temperature to 37.0°C, the normalization of the skin colour, and the reduction of a pathological capillary pattern area on the skin of the lower limbs. Later, we observed the gradual recovery of vital organ functions, passage through the intestine, and renal excretory functions against the background of the ongoing standard multicomponent treatment for sepsis and MODS.

The analysis of this clinical case allows for the conclusion that, despite the low value of the endotoxin activity level, the extremely low «Response» level with high PSP, PCT, and CRP values may be an absolute indication for selective LPS-adsorption.

## 4. Discussion and Conclusions

Septic complications in cardiac surgery remain a serious problem that require the development of timely diagnosis and treatment strategies. EAA is fully automated, minimizing the frequency of random and systematic errors, at both the stages of collecting and interpreting the results. Endotoxin activity measurement in the EAA is directly related to the phagocytic ability of neutrophils, opsonized with immune complexes, “endotoxin-endotoxin antibodies,” and a resultant respiratory burst, the intensity of which is automatically fixed by the chemiluminometer [14]. The principle of a chemiluminescence measurement of phagocytic activity was proposed by Allen R.C. in 1986. It is based on CR1/CR3 receptor activation of neutrophils in the whole blood, which produce oxidants that react with luminol [19].

Among the examined patients who were enrolled in the study, the observed “Response” value corresponded to adequate neutrophil phagocytic activity. In the group with critically low “Response,” the low EAA value should be noted (Table 2). Moreover, this group had the highest presepsin concentration (*p* < 0.05). Presepsin is a soluble CD14 subtype, which is cleaved from the monocyte after binding with the endotoxin when phagocytosis activation occurs [20].

The analysis of the published data and the results obtained in this study allowed for the proposal of the following hypothesis. The prolonged adsorption of massive endotoxin doses into the bloodstream causes a high phagocytic activity within the body, which reflects the significantly increased presepsin concentration, leading to the inability of the patient's neutrophils to react with the immune complexes in the EAA test as their CR1/CR3-receptors are busy or exhausted by the prolonged activity.

Data from current studies confirm that the suppression of immune responses during inflammation and severe sepsis induces the same mechanisms involved in the adequate antimicrobial body's response [21]. The aberrant control (associated with nonresolving inflammation or a secondary bacterial invasion) of such mechanisms produces the results as follows: the auto- and paracrine cleavage of receptors on the surface of the PMN, the reduction of surface receptor expression levels and inhibition of signalling pathways, and the appearance of immature neutrophils with a reduced phagocytic activity in the systemic circulation [21].

In our case, the decrease in neutrophil response is most likely due to both the high concentration of microorganisms and, therefore, a high share of the internalization of CR3-receptors and the possible splitting of neutrophil CR1-receptors in the presence of high levels of serine proteases [22, 23].

The group with a critically low “Response” has also been noted to have the highest D-dimer concentration (*p* < 0.05), which is the end-product of cross-linked fibrin degradation and is within the scope of the proposed hypothesis (Table 2). Neutrophil granules contain multiple hydrolytic enzymes, which can have a devastating effect on a plurality of different protein substrates, including fibrin and during the excessive secretion of phagocytic neutrophils [24]. The leading role in the destruction of the protein-native structure belongs to serine protease because it shows broad substrate specificity. This group also includes the elastase enzymes involved in the cleavage of a soluble CD14, the product of which is presepsin [25]. Another verification of the proposed hypothesis, based on the dysfunction of neutrophil receptors in septic patients, is the nonsignificant differences in WBCs and the proportion of phagocytic subpopulations between the study groups with different “Response” levels (Tables 2 and 3).

One can judge the practical confirmation of our hypothesis according to the results of the above clinical case of the septic patient with a critically low “Response.” After a second LPS-adsorption procedure, the ability of the patient's neutrophils to resist the oxidative burst increased to 37%, and the value of endotoxin activity reached the upper limit of the range that could be associated with the release of PMNs by CR1/CR3-receptors due to reducing the endotoxin level. It should be noted that all the septic patients undergoing LPS-adsorption demonstrated positive dynamics of neutrophil response.

Thus, EAA values should be interpreted considering the level of “Response,” which has a negative predictive value when it is lower than 0.5. The EAA values < 0.6 at “Response” ≥ 0.5 indicate the absence of systemic endotoxaemia in a patient's blood stream, whereas EAA values < 0.6 at “Response” < 0.5 correspond to the impossible exclusion of an increased endotoxaemic level.

To make a decision in Bakulev NSPCCS we use the data of procalcitonin and Endotoxin Activity Assay (EAA) levels as the indications for the LPS-adsorption. The results obtained in this study demonstrate the necessity of including both the “Response” and the PSP assessment when prescribing a panel of laboratory tests and monitoring pathogenic therapy in patients with sepsis. This proposed algorithm will help to create a more complete and accurate assessment of the patient's clinical status in each definitive level of studying endotoxaemia, which will increase the informative value of the panel of laboratory tests and the effectiveness of the therapy in general. Our results help to diagnose high levels of endotoxaemia even in a difficult case and to indicate the necessity in using the LPS-adsorption. It leads to the increased survival of septic patients with reduced immune reactivity.

## Figures and Tables

**Table 1:** Patient characteristics.

Parameters	Values, Me (P_25_; P_75_)
Age, years	57 (46; 63)
Gender, m/f	123/75
Weight, kg	78 (70; 88)
CPB, min	190 (143; 252)
Myocardial ischaemia, min	109 (80; 148)
Body temperature, °C	38,3 (37,8; 38,9)
MAP, mmHg	71 (65; 85)
HR, beats/min	97 (90; 110)
APACHE II	26 (19; 30)
SOFA	12 (10; 14)
Epinephrine, ug/kg/min	0.076 (0.050; 0.120)
Norepinephrine, ug/kg/min	0.17 (0.05; 0.29)
Dopamine, ug/kg/min	5.0 (5.0; 7.0)
PaO_2_/FiO_2_	223 (152; 289)

**Table 2:** Inflammatory and endotoxaemia markers in patients with different «Response» levels.

Parameter	Group 1 (*n* = 164) 1.00–0.80	Group 2 (*n* = 24) 0.79–0.50	Group 3 (*n* = 10) 0.49–0	P_1–3gr_
“Response”	093 (0.88; 0.95)	0.65 (0.57; 0.76)	0.38 (0.30; 0.49)	—
EAA	0.62 (0.47; 0.70)	0.66 (0.38; 0.87)	0.34 (0.31; 0.72)	0.07
CRP, mg/dL	9.9 (5.5; 15.9)	12.0 (6.9; 19.0)	9.5 (6.3; 15.8)	0.63
PCT, ng/ml	5.9 (2.4; 16.2)	8.4 (2.1; 17.8)	4.9 (2.0; 8.8)	0.83
PSP, pg/ml	2191 (1092; 3782)	1808 (986; 7124)	9974 (4653; 14187)	**0.05**
	**P**_**gr**1/**gr**3_ = 0.01	**P**_**gr**2/**gr**3_ = 0.05		
WBC, *∗*10^9^/L	14.8 (10.2; 23.7)	15.2 (10.7; 21.9)	11.2 (9.4; 16.0)	0.35
D-dimer, ng/ml	813 (416; 2324)	612 (324; 1530)	2664 (807; 4560)	**0.05**
	**P**_**gr**1/**gr**3_ = 0.05	**P**_**gr**2/**gr**3_ = 0.04		

**Table 3:** Proportion of phagocytic leukocyte subpopulations in patients with different «RESPONSE» levels.

Parameter	Group 1 (*n* = 164) 1,00–0,80	Group 2 (*n* = 24) 0,79–0,50	Group 3 (*n* = 10) 0,49–0	P_1–3gr_
Neutrophils, %	87 (72; 95)	83 (75; 94)	83 (73; 93)	0.74
Monocytes, %	4 (3; 5)	5 (3; 6)	5 (3; 8)	0.33
Eosinophils, %	1 (1; 2)	3 (2; 4)	2 (2; 3)	0.56

**Table 4:** Inflammatory and endotoxaemia markers in septic patients undergoing LPS-adsorption.

Parameter	*n* = 66
“Response”	0.90 (0.83; 0.94)
EAA	0.73 (0.66; 0.80)
CRP, mg/dL	11.5 (7.1; 19.9)
PCT, ng/ml	6.2 (3.0; 16.2)
PSP, pg/ml	2598 (1416; 4761)
WBC, *∗*10^9^/L	14.2 (10.9; 19.6)
D-dimer, ng/ml	974 (494; 3234)

**Table 5:** Dynamics of laboratory parameters after two selective LPS-adsorption procedures.

Parameter	Before LPS-adsorption	After LPS-adsorption
«Response»	0.01	0.37
EAA	0.00	0.99
CRP, mg/dl	5.8	7.5
PCT, ng/ml	5.5	8.7
PSP, pg/ml	9482	8367
WBC, *∗*10^9^/L	3.0	11.0
D-dimer, ng/ml	7472	6086

## Abbreviations

APACHE II: Acute physiology and chronic health evaluation score
NYHA: New York Heart Association score
SOFA: Sequential organ failure assessment score
MODS: Multiple organ dysfunction syndrome
SIRS: Systemic inflammatory response syndrome
EAA: Endotoxin Activity Assay
ICU: Intensive care unit
LPS: Lipopolysaccharide
LVEF: Left ventricle ejection fraction
CRP: C-reactive protein
PCT: Procalcitonin
PSP: Presepsin
PMX: Polymyxin B cartridge
SIRS: Systemic inflammatory response syndrome
WBC: White blood cells
PMN: Polymorphonuclear neutrophils.
